# Current Therapy in CKD Patients Can Affect Vitamin K Status

**DOI:** 10.3390/nu12061609

**Published:** 2020-05-30

**Authors:** Mario Cozzolino, Giuseppe Cianciolo, Manuel Alfredo Podestà, Paola Ciceri, Andrea Galassi, Lorenzo Gasperoni, Gaetano La Manna

**Affiliations:** 1 Renal Division, ASST Santi Paolo e Carlo, Department of Health Sciences, University of Milan, 20142 Milan, Italy; mario.cozzolino@unimi.it (M.C.); manuel.podesta@unimi.it (M.A.P.); andrea.galassi@asst-santipaoloarlo.it (A.G.); 2 Nephrology, Dialysis and Renal Transplant Unit, Department of Experimental Diagnostic and Specialty Medicine (DIMES), S. Orsola Hospital, University of Bologna, 40126 Bologna, Italy; giuseppe.cianciolo@aosp.bo.it (G.C.); lorenzo.gasperoni3@gmail.com (L.G.); 3 Renal Research Laboratory, Department of Nephrology, Dialysis and Renal Transplant, Fondazione IRCCS Ca’ Granda, Ospedale Maggiore Policlinico, 20122 Milan, Italy; p.ciceri@hotmail.it

**Keywords:** vitamin K, chronic kidney disease, vascular calcification, secondary hyperparathyroidism, warfarin

## Abstract

Chronic kidney disease (CKD) patients have a higher risk of cardiovascular (CVD) morbidity and mortality compared to the general population. The links between CKD and CVD are not fully elucidated but encompass both traditional and uremic-related risk factors. The term CKD-mineral and bone disorder (CKD-MBD) indicates a systemic disorder characterized by abnormal levels of calcium, phosphate, PTH and FGF-23, along with vitamin D deficiency, decreased bone mineral density or altered bone turnover and vascular calcification. A growing body of evidence shows that CKD patients can be affected by subclinical vitamin K deficiency; this has led to identifying such a condition as a potential therapeutic target given the specific role of Vitamin K in metabolism of several proteins involved in bone and vascular health. In other words, we can hypothesize that vitamin K deficiency is the common pathogenetic link between impaired bone mineralization and vascular calcification. However, some of the most common approaches to CKD, such as (1) low vitamin K intake due to nutritional restrictions, (2) warfarin treatment, (3) VDRA and calcimimetics, and (4) phosphate binders, may instead have the opposite effects on vitamin K metabolism and storage in CKD patients.

## 1. Introduction

Cardiovascular (CV) disease is the most common cause of morbidity and mortality in patients with chronic kidney disease (CKD) [1,2].

The high CV risk may be due, at least in part, to the excess of vascular calcifications (VC) observed even in very young dialysis patients, who lack the typical CV risk factors such as hypertension, dyslipidemia, and smoking [3,4,5]. The presence of abdominal aortic calcifications is significantly associated with all-cause and cardiovascular mortality in maintenance hemodialysis (HD) patients [2].

Although arterial medial calcification is the most represented, both intimal and medial calcifications coexist in CKD patients [3]. Medial and intima calcification are active processes that share some triggering factors: mineral bone disorders, inflammatory status, humoral factors, and phenotypic switch of resident or circulating precursor [3,6,7,8,9,10]. Chronic kidney disease–mineral bone disorder (CKD-MBD) is considered one of the main factors associated with increased cardiovascular morbidity and mortality in CKD patients [11]. These patients frequently show both impaired bone mineralization and ectopic vessel mineralization, a feature which is the result of at least two factors: the “bone–vessel axis” and the “calcification paradox”.

Albeit some pathways involved in the crosstalk between vasculature and bone (the “bone-vascular axis”) are still unknown, it is clear that osteotropic hormones, including parathyroid hormone and calcitriol, physiologically regulate both vascular and skeletal mineralization as well as stem cell renewal [9,12]. Cellular, endocrine and metabolic signals that flow bidirectionally between vasculature and bone are necessary for the health of both. Diabetes and CKD in particular are associated with an important derangement of the bone–vascular axis which usually determines simultaneous alterations in both.

VC is frequently associated with and evolves in parallel with decreased bone mineral density and deranged bone turnover. Bone and vessel mineralization share common pathways, and this parallel is known as the “calcification paradox” [13]. Moreover, treatments targeting bone disorders might have adverse consequences for cardiovascular health.

This creates the need to identify treatments that have a positive impact on the factors or pathways involved in the crosstalk between vasculature and bone. Over the last decade, as a growing body of evidence has seemed to point to an involvement of vitamin K deficiency in VC, the hypothesis that vitamin K supplementation could be a tool to prevent the rapid progression of VC in CKD patients is gaining momentum [14].

## 2. Vitamin K and Gla Proteins

There are two main natural forms of Vitamin K: K1 (or phylloquinone, PK) contained in green vegetables, and K2 (including several different vitamers called menaquinones, MKs) mostly derived from fermented foods and intestinal bacteria (e.g., cheeses and the Japanese soybean product known as “natto”) [15]. There are up to 12 different types of MKs, from MK-4 to MK-15; the most common MKs in humans are the short-chain MK-4 and MK-7 to MK-10, which are respectively produced by systemic conversion of phylloquinone to menaquinones and synthesized by bacteria [16]. The pivotal biological role of vitamin K is to act as a cofactor for the carboxylation (and thereby activation) of vitamin-K-dependent proteins (VKDPs). Vitamin-K-dependent carboxylase is an enzyme that catalyzes the addition of carbon dioxide to the specific glutamic acid residues of a limited number of proteins leading to the formation of g-carboxyglutamic (Gla) residues.

Seventeen members of the Gla protein family, involved in various biological processes, have currently been identified. In particular, the Gla family includes (i) seven proteins belonging to the coagulative cascade: prothrombin, factor VII, factor IX, factor X, protein C, protein S and protein Z; (ii) four proteins that regulate bone and vascular mineralization: matrix Gla protein (MGP), osteocalcin (OC), growth arrest-specific protein 6 (Gas6), Gla-rich protein (GRP); (iii) two proline-rich Gla proteins, two transmembrane Gla proteins, periostin and periostin-like factor [17,18,19,20].

Measuring Vitamin K plasma levels is difficult due its low circulating concentration, the non-polar nature of the molecule and the interference of lipids. Moreover analytical variability, diet, inflammation, and the coexistence of chronic disease may further influence plasma levels of vitamin K subtypes [21]. Functional tests can be performed to estimate vitamin K blood levels indirectly. In particular, the measurement of undercarboxylated proteins, OC and MGP, has proved to be more sensitive than prothrombin time in detecting subclinical vitamin K deficiency. Vitamin K deficiency status does not allow VKDPs to acquire their carboxylated form: the gamma-carboxyglutamate (Gla) domain is responsible for the high-affinity binding of calcium ions, thus allowing coagulation factors OC and MGP to interact with negatively charged phospholipid membranes. Moreover, adequate calcium binding is a critical physiologic step in bone mineralization counteracting VC [22].

Since some Gla-proteins are involved in bone metabolism and vascular health, their reduced carboxylation leads to bone metabolism impairment and an increase in the VC formation process [23]. Mild vitamin K deficiency does not usually induce clinically evident changes in coagulation, which is consistent with the observation that functional tests (e.g., prothrombin time) are altered only when the activity of vitamin K-dependent coagulation is reduced by 50% [24,25]. On the other hand, due to differences between hepatic and extra-hepatic vitamin K metabolism, extra-hepatic VKDP carboxylation is most affected by subclinical deficient states [25]. The current understanding is that vitamin K2 is mainly transported to the extra-hepatic tissues and controls the carboxylation of VKDPs in bone and blood vessels, while vitamin K1 is central for the carboxylation of vitamin-K-dependent coagulation factors in the liver [25].

Considering that each Gla-protein has specific biological functions, no single biomarker is considered a gold standard in the assessment of vitamin K deficiency.

OC is synthesized by the osteoblasts under the control of vitamin D, and its active carboxylated form (cOC) is mainly involved in bone mineralization, since it binds and incorporates calcium ions in the hydroxyapatite crystals in bone matrix. Thus, a high undercarboxylated OC (ucOC) level is an expression of poor vitamin K levels and intake. Moreover ucOC can be released during osteoclastic resorption, and is thought to be responsible for functions related to energy metabolism [17,26].

Low levels of ucOC are associated with higher bone mineral density and reduced fracture risk [27]. It is a fact that ucOC mirrors total OC, and expressing ucOC as a percentage of the total OC (%ucOC) may therefore be considered a more reliable index of vitamin K storage than the absolute value of ucOC. As showed by Booth et al., a value of %ucOC >20% is congruent with subclinical vitamin K deficiency in vitamin K depletion and repletion studies [28].

As OC distribution seems to change with age and gender, reflecting the degree of bone formation, the differences in its distribution in bone matrix may be responsible, at least in part, for the altered remodeling of bone associated with gender and aging [29,30,31]. In addition, glutamic acid carboxylation at position 17 is probably fundamental for the spatial and structural conformation of the osteocalcin, allowing a correct interaction with hydroxyapatite crystals [18].

It has been observed that mice knocked out for the OC gene are affected by hyperostosis, suggesting that osteocalcin is crucial for bone formation. Vitamin K2 seems not to be involved only in OC carboxylation, but also in the reduction of bone resorption through the increase in osteoprotegerin (OPG) production and the inhibition of RANK ligand (RANKL) expression [32]. MGP is a 12-KDa gamma-carboxyglutamic acid-containing protein synthesized by vascular smooth muscle cells (VSMC) and chondrocytes; it is currently considered one of the most potent endogenous inhibitors of vascular calcification in vivo [33,34,35].

The protein undergoes two types of post-translational modification before maturation: (i) phosphorylation of up to three serine residues and (ii) gammacarboxylation of up to five glutamate residues. While the role of carboxylation, which depends on Vitamin K, is better understood and determines MGP’s bioactivity as a calcification inhibitor, the function of phosphorylation is not yet clarified; recent data, however, suggest a role in regulating MGP secretion into the extracellular milieu [36,37]. The aforementioned reactions do not proceed in parallel, and, according to the state of carboxylation and/or phosphorylation, at least four different molecules can be found in circulation: (i) dephosphorylated uncarboxylated MGP (dp-ucMGP); (ii) dephosphorylated carboxylated MGP (dp-cMGP); (iii) phosphorylated uncarboxylated MGP (p-ucMGP); (iv) phosphorylated carboxylated MGP (p-cMGP). The active form is both phosphorylated and carboxylated (p-cMGP) and its synthesis is stimulated by vitamin D [38].

Findings about ucMGP levels in the bloodstream of CKD patients are contradictory. Low, rather than high, circulating ucMGP levels were a powerful predictor of cardiovascular calcification, and ucMGP was decreased within the serum of CKD patients: this feature could reflect an accumulation of ucMGP within the calcified vessel wall [39,40]. Krueger speculated that uncarboxylated Gla proteins are less secreted from the cells, maybe in attempt to prevent inactive proteins from entering the tissues. A reduced phosphorylation of the three serine residues of MGP seems equally important as it would result in reduced secretion of MGP [16,17,18,19,36,41].

The fully inactive form of the protein, dephosphorylated uncarboxylated MGP (dp-ucMGP), does not interact with calcium or BMP-2 and closely reflects the vitamin K status at the vascular level [42,43], thus representing a potential biomarker for cardiovascular endpoints. Dp-ucMGP levels show an inverse correlation with vitamin K status, hence decreasing with vitamin K supplementation and increasing with vitamin K antagonist [44].

True deficiency and functional shortage of Vitamin K (as occurs in the case of warfarin treatment) through the reduction of active MGP and OC may therefore lead to vascular calcification and impaired bone mineralization.

Some members of the Gla protein family (GRP and MGP) are components of circulating calciprotein particles (CPP) and extracellular vesicles (EVs) in which they play an important role in regulating the calcification process. Circulating CPP are colloidal nanoparticles diffused in the blood and mainly composed of Ca and P precipitates, fetuin-A, GRP and probably MGP. They are considered a possible mechanism by which ectopic mineralization is prevented. Fetuin, GRP, and other soluble proteins contained in CPP act like mineral carriers with a role in the stabilization, transport and recycling of water-insoluble minerals in the blood. A pivotal event in the onset of VSMC calcification is the release of extracellular EVs capable of efficiently nucleating hydroxyapatite in the absence of calcification inhibitors like MGP. It is likely that γ-carboxylated GRP, fetuin- A, and MGP represent an effective mechanism to regulate the steps of mineral formation both at systemic and tissue levels, thus preventing pathological calcification [20,45,46]. If the content of fetuin-A, GRP or MGP is insufficient or Gla proteins are predominantly undercarboxylated, CPPI would be transformed to crystalline mineral core (CPPII) that cannot be excreted through the liver [20]. A high proportion of CPPII induces endothelial damage and, by deposition in the extracellular matrix, an enhancement of VC [20,47].

Therefore, an insufficient level of calcification inhibitors, or a lower activation of Gla-proteins, whatever the cause, predisposes to vascular calcification [47,48]. The subsequent release of extracellular vesicles, containing less fetuin-A or GRP, could play an additional role in modulating the VSMC calcification process [20,49].

## 3. Vitamin K Deficiency, MGP and OC Metabolism in CKD Patients

Observational and interventional clinical studies suggest that CKD patients often develop subclinical vitamin K deficiency [50,51,52], a condition that may determine a higher risk of morbidity and mortality in this population. Dietary recommendations aimed at restricting potassium and phosphate intake (e.g., reduced consumption of leafy green vegetables and dairy products, rich in vitamin K1 and K2, respectively) may play a pivotal role in promoting this deficit [17,21,50,53].

Vitamin K deficiency is also supposedly due to the uremic milieu and to the exhaustion caused by the high volume of vitamin K required by VKDPs to inhibit calcification.

CKD patients are exposed to the risk of subclinical Vitamin K deficiency, which entails a reduced activation of MGP and OC, both playing a crucial role in vascular and bone health. Thus, it is not unexpected that in CKD patients Vitamin K deficiency can contribute to the high VC burden as well as to the derangement of bone metabolism.

However, in CKD, especially in patients that are not on dialysis, studies on the effect of vitamin K deficiency on OC (expressed as ucOC or %ucC cut off) and bone health are still limited.

MGP could counteract the progression of vascular calcification in CKD patients by binding hydroxyapatite crystals; this interferes with their deposition and promotes macrophage-mediated clearance [19]. In addition, the interaction between active MGP and bone morphogenetic protein-2 (BMP-2) results in the inhibition of VSMC osteoblast transformation [54,55], a central mechanism in the development of vascular calcification [56].

Vitamin K deficiency leads to the formation of uncarboxylated MGP (ucMGP), whose concentration on arterial walls progressively increases along with the severity of the vascular calcifications [57]. Deposits in the blood vessels could explain the low circulating ucMGP levels usually found in patients with end-stage renal failure on chronic hemodialysis [40,58,59], and may provide the rationale for the inverse relationship between ucMGP serum concentration and vascular calcification observed in some of these studies.

In CKD patients, an increase in the fully inactive form of MGP, dp-ucMGP, has been shown in CPP1 and matrix vesicles. This entails a faster transformation of CPP1 into CPP2, and this accelerated transformation is associated with VC [48]. Moreover, a reduced amount of fetuin-A and active GRP has been related to a more severe mineral calcification in the soft tissue [20].

The concentration of circulating dp-ucMGP progressively increases across CKD stages [38,60,61]; its levels have been associated with the severity of aortic calcifications [38,61,62] and vascular stiffness [63,64] in many studies, albeit not all (Table 1) [43,65]. Moreover, high dp-ucMGP represents an independent predictor of mortality in renal transplant recipients [66] but not in other groups of CKD patients [38,43]. Interestingly, dp-ucMGP levels in blood [67] and kidney tissue [68] also predict the impairment of renal function both in the general population and in CKD patients, suggesting that vitamin K deficiency may increase the risk of progression towards end-stage renal disease. Even though vitamin K2 supplementation can lower dp-ucMGP levels in patients with CKD [43,69,70,71], the effects of this treatment on clinical outcomes still need to be fully clarified.

In CKD patients, vitamin K levels can be further influenced by the frequent use of the anticoagulant warfarin [72]. Warfarin is a 4-hydroxycoumarin derivative that selectively inhibits the oxidoreductase responsible for the regeneration of inactive and dietary vitamin K, thus preventing the hepatic gamma-glutamyl carboxylation of coagulation factors [73]. In the liver, however, the recycling of inactive vitamin K also depends on the activity of DT-diaphorase, a reducing enzyme not affected by warfarin, that activates vitamin K and thus limits the effect of the drug. On the other hand, DT-diaphorase activity is extremely low in VSMC and other extra-hepatic tissues [74]. Thus, the carboxylation of extra-hepatic VKDP, including MGP, heavily relies on the activity of vitamin K oxidoreductase, and is therefore more susceptible to warfarin treatment.

Consistently with these data, simultaneous administration of warfarin and vitamin K to Wistar Kyoto rats does not affect the synthesis of coagulation factors but blocks MGP carboxylation, inducing diffuse medial vascular calcification, which can be only partially relieved by a vitamin K-rich diet [38]. Similarly, in an animal model of CKD, a lower therapeutic dose of warfarin has promoted calcium deposition in major blood vessels and induced arterial stiffening [75]. Interestingly, the same treatment did not induce medial calcification in rats without kidney dysfunction, suggesting that vitamin K deficiency potentiates the effect of other pro-calcifying factors in CKD patients. In keeping with experimental evidence, administration of warfarin in CKD patients has been associated with a steep increase in the concentration of circulating dp-ucMGP, while stopping the treatment has consistently reduced dp-ucMGP levels [44,62,76].

From a clinical standpoint, patients on hemodialysis treated with warfarin had a 3.77 odds ratio of developing severe aortic calcifications compared to those not taking the drug [77]. Long-term warfarin use was also associated with increased coronary and extra-coronary calcifications [78,79,80]. A cross-sectional study across 1838 Japanese dialysis centers identified warfarin use as an independent predictor of calciphylaxis (i.e., calcific uremic arteriolopathy) [81], a severe complication of end-stage renal disease. These data raise concerns about the risk–benefit ratio of the use of warfarin in the hemodialysis setting for conditions such as atrial fibrillation; indeed, a retrospective cohort study of 41,425 patients on hemodialysis highlighted that warfarin use was associated with a higher mortality in this group, even after stratification and covariate adjustment [82]. The same group also reported an increased risk of stroke in hemodialysis patients with pre-existing atrial fibrillation treated with warfarin compared to those not assuming the drug. This suggests that, in this setting, warfarin might not be as beneficial as in the general population [83].

Several studies show that hemodialysis patients’ low vitamin K levels are likely related to the dietary regimen prescribed and to overall poor nutritional intake. Moreover, given its lipophilic characteristics, vitamin K is not supposed to be removed or absorbed by the dialysis membrane.

Cranenburg et al. evaluated the vitamin K1 and K2 intake and the vitamin K status of 40 haemodialysis patients and found a low intake (median 140 μg/day) in dialysis per day and during the weekend, compared to healthy adults (mean K1 and K2 intake 200 and 31 μg/day, respectively).. Undercarboxylated VKDPs (dp-ucMGP and ucOC) were found to be high in 33 HD patients, pointing to hepatic as well as extrahepatic deficient vitamin K status. On the other hand, high non-carboxylated MGP in the same population strongly suggested vascular vitamin K deficiency [52]. Voong et al. reported that 29% and 93% of maintenance HD patients met the criteria for sub-clinical vitamin K deficiency based on low levels of phylloquinone and high levels of ucOC, respectively, suggesting vitamin K deficiency at the bone level [51]. Westenfeld et al. demonstrated that haemodialysis patients had 4.5-fold higher dephosphorylated, uncarboxylated MGP and 8.4-fold higher uncarboxylated osteocalcin levels compared with controls, confirming that most hemodialysis patients have a functional vitamin K deficiency [71]. Fusaro et al. carried out an observational study to assess the prevalence of vitamin K deficiency and the relationship between vitamin K status, vertebral fractures, vascular calcification, and survival in 387 patients on hemodialysis for ≥1 year. Total OC and ucOC levels were higher in patients with CKD than in healthy controls, and vitamin K1 deficiency was an independent predictor for vertebral fractures in prevalent hemodialysis patients (the median total OC level was 29% lower in patients with one or more vertebral fractures) [84].

Peritoneal dialysis (PD) patients and maintenance HD patients displayed a similar degree of vitamin K depletion. The results of a cross-sectional study carried out on 21 PD patients demonstrate that a significant proportion of patients have subclinical vitamin K deficiency, defined by low serum phylloquinone concentrations (<0.4 nmol/L) and elevated %ucOC (>20%), respectively, at 23.8% and 100% [85].

Jansz et al. analyzed the effect of kidney transplantation and phosphate binder use on vitamin K status; they found lower dp-ucMGP levels in kidney transplant recipients compared to patients on HD or peritoneal dialysis, which seem to suggest vitamin K levels improve after kidney function is restored. An important exception is represented by sevelamer monotherapy, which is associated with significantly higher dp-ucMGP levels; this suggests that sevelamer has a negative effect on patients’ vitamin K status [86].

## 4. Effects of CKD-MBD Therapy on Vitamin K and Vitamin K Dependent Proteins

The mainstays of treatment of CKD-MBD are (i) lowering increased parathyroid hormone levels with vitamin D receptor activators (VDRA) and/or calcimimetics and (ii) decreasing serum phosphorus level with dietary interventions and phosphate binders. However, due to the wide variation in serum levels of mineral metabolism markers and to the variability of therapeutic response among patients, CKD-MBD therapy needs to be tailored to the individual patient.

Calcifediol (25OH) is the precursor of calcitriol and is regarded as the best indicator of vitamin D status, although a significant number of studies have shown no effect on parathyroid hormone (PTH) suppression; in experimental studies performed in calcifediol-deficient mice, calcifediol administration reduced the extent of calcification, but did not induce any changes in systemic calcium and phosphate levels [87].

Actually, calcitriol may cause detrimentally elevated levels of serum calcium and phosphate [88]. At high doses, it activates specific vitamin D receptors (VDR) expressed in vascular smooth muscle cells (VSMCs), and promotes VC both in vitro and in vivo, while at more physiological doses vitamin D could even be protective [3,89,90]. These results suggest that vitamin D derivatives have different effects on calcification. In this scenario, the active vitamin D metabolite may favor the calcification process through the stimulation of calcium and phosphate, while its precursor form (calcifediol) may be able to provide protection against calcification via calcium- and phosphate-independent mechanisms [3,91]. Calcimimetics, by binding with calcium-sensing receptors, increase the sensitivity to extracellular calcium, lowering PTH synthesis and secretion. Unlike VDRA, they do not increase calcium and phosphorus levels and prevent VC [92]. Fusaro et al. performed a secondary analysis [84] of the VIKI (VItamin K Italian) study, an observational study designed to assess the prevalence of vitamin K deficiency in hemodialysis patients and to investigate the effects of ongoing treatment for CKD-MBD on OC and MGP levels. They showed that vitamin D analogs increase the levels of OC and MGP, while calcimimetics, alone or combined with calcium acetate, increase only MGP levels; in addition, the combination of vitamin D analogs and calcimimetics proved to be most effective in inducing a further increase of total OC [93], while increased total MGP levels are found only in patients treated with calcimimetics, alone or combined with calcium acetate [93]. Moreover, in this study, lower OC levels were associated with vertebral fractures or vascular calcification, while higher baseline total OC levels were combined with a lower progression rate of abdominal aortic calcification; OC thus appears to protect against VC, although it promotes mineralization of bone tissue. Although it is likely that the effect on OC and MGP of both vitamin D analogs and calcimimetics may be indirectly mediated by the PTH decrease, it cannot be excluded that a direct gene/transcriptional (direct) effect may be at work not only for vitamin D analogs but also for calcimimetics [93].

Therefore, patients with CKD and hyperparathyroidism could disclose low serum OC, but this does not necessarily entail that they are vitamin-K-deficient, and could be the expression of the PTH activity. In CKD patients, hyperphosphatemia is commonly treated with dietary restrictions and intestinal phosphorus binders. A range of phosphate binders currently available for long-term use include calcium-based phosphate binders (calcium carbonate and acetate) and calcium-free binders (aluminum hydroxide, lanthanum carbonate, magnesium carbonate, sevelamer hydrochloride, and sevelamer carbonate). As current phosphate binders are all effective in lowering phosphorus, the main considerations for selecting one over another include absorbability (ideally non-absorbed), compliance, adequate gastrointestinal tolerability, and cost or cost-effectiveness.

Is not yet clear weather calcium-based phosphate binders are associated with harmful effects. Non-calcium-based phosphate binders demonstrate a reduction of VC progression and significant survival benefits over a 3-year interval compared to calcium-based ones [94,95,96,97,98]. However, the results in this regard are contradictory [99,100].

VC frequently progress despite adequate phosphate levels, probably following chronic inflammation, increased calcium load from calcium-based binders and, not least, the capacity of phosphate binders to also bind vitamin K. Therefore, the advantage of lowering phosphate blood levels may be blunted by a worsening of vitamin K deficiency, a possible off-target effect of phosphate-binding therapy. Studies looking at this problem are still limited, and, most importantly, the results show noticeable differences in the association between various phosphate binders and vitamin K deficiency.

Neradova et al. evaluated the interaction of vitamin K2 (menaquinone-7; MK-7) with five different phosphate binders, in the presence or absence of phosphate [101]. In this in-vitro study sucroferric-oxyhydroxide and sevelamer carbonate were the only binders that did not interact with vitamin K2. Instead, calcium acetate/magnesium carbonate bound vitamin K2 strongly, both in the absence and presence of phosphate. The binding of lanthanum carbonate and vitamin K2 depended on the absence of phosphate, suggesting a competitive interaction between phosphate and vitamin K2 for this compound. No significant binding was observed in the solution containing vitamin K2 and phosphate. Calcium carbonate significantly bound vitamin K2 in a solution containing phosphate, while no significant binding was observed without phosphate. A nominally lower concentration of K2 was shown in the mixture with sevelamer carbonate as well, but this decline was not statistically significant. Finally, the addition of sucroferric-oxyhydroxide did not lead to any decline of vitamin K2, irrespective of the presence or absence of phosphate.

**Figure 1:**
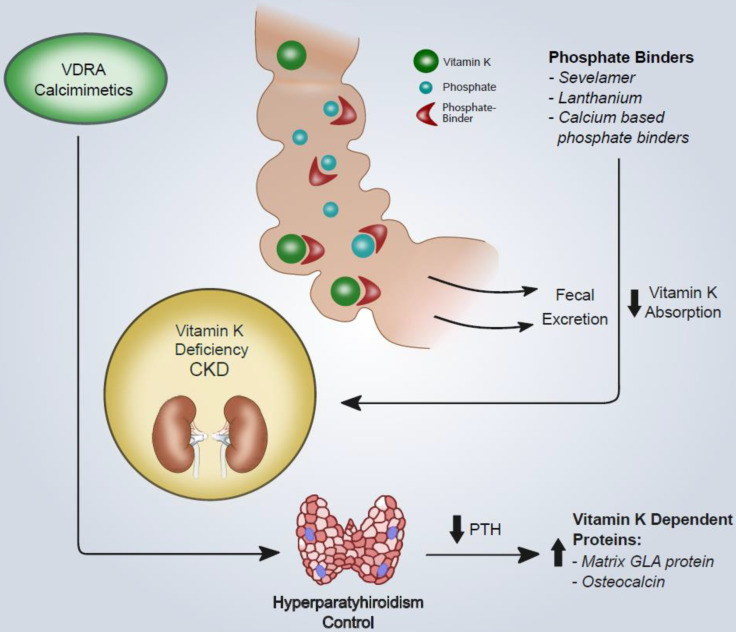
Overview of the effects of CKD-MBD therapies (VDRA, calcimimetics and phosphate binders) on vitamin-K-dependent protein activation and intestinal vitamin K adsorption. VDRA and calcimimetics increase the levels of both OC and MGP. Although the exact mechanism is still unknown, such an effect could be mediated by PTH decrease. However, a direct gene/transcriptional effect may be present not only for vitamin D analogues but also for calcimimetics. Phosphate binders may also bind vitamin K. The interaction between any phosphate binder and vitamin K2 depends on the specific physical and chemical properties of any binder. Abbreviations: VDRA, vitamin D receptor agonist; CKD, chronic kidney disease; PTH, parathormone.

The results of this study suggest that: (i) the interaction between any phosphate binder and vitamin K2 depends on the specific physical and chemical properties of the binder; (ii) there is no direct interaction between vitamin K2 and phosphate; (iii) only in the case of calcium carbonate can the phosphate facilitate the link of this binder with vitamin K2 (Figure 1).

Sevelamer carbonate, because of its binding to bile acids, was expected to impair the absorption of fat-soluble vitamins like vitamin K2. However, Westenfeld et al. [71], in agreement with the findings by Neradova et al. [101], found no association between sevelamer administration and vitamin K (menaquinone) levels, although only a small proportion of patients were prescribed sevelamer. On the contrary, another in-vitro study shows extensive vitamin K binding by sevelamer hydrochloride [102].

## 5. Conclusions

Although several studies seem to assess the involvement of functional vitamin K deficiency in the pathogenesis of vascular calcifications, as yet there are no studies in CKD and dialysis population demonstrating that vitamin K supplementation can prevent the development of vascular calcifications as well as the associated cardiovascular morbidity and mortality.

Undercarboxylated Gla-proteins are used as markers for vitamin K levels in the tissues from which they are produced, and their high levels are considered equivalent to functional vitamin K deficiency, although the threshold below which vitamin K status can be considered optimal is not yet known. A significant contribution to the understanding of the role of vitamin K deficiency could derive from future studies on vitamin-K-dependent GRP and calcification propensity score (T50) [103].

The current understanding is that vitamin K deficiency in CKD patients is mainly related to dietary recommendations, while the exact role of the uremic state itself, and the high demand of vitamin K in the “pro-calcifying” uremic environment, remain unknown (Table 1). Future studies will better define the effects of ongoing treatment for mineral and bone disorders on OC and MGP metabolism.

## Figures and Tables

**Table 1:** Association between vitamin K status and cardiovascular outcome in CKD populations.

Study	Design/Patients	Vitamin K Assessment	Outcome	Results
Puzantian et al., 2018 [64]	Prospective 137 CKD 1–5	dp-ucMGP	dp-ucMGP CF-PWV	Increase with CKD severity (*p* < 0.0001) Positive correlation (β = 0.21; *p* = 0.019).
Thamratnopkoon et al., 2017 [61]	Cross-sectional 83 CKD 3-5	dp-ucMGP	Vascular Calcification Score (Kauppila)	OR 1002, *p* = 0.004
Kurnatowska et al., 2016 [60]	RCT, DB 38 CKD 4–5	dp-ucMGP	dp-ucMGP after Vit K2 for 9 m	Decreased dp-ucMGP Vs control (10.7% vs. no change)
Meuwes et al., 2015 [65]	Cross-sectional 64,9% on Dialysis	dp-ucMGP PIVKA-II	CAC Score	No association
Delanaye et al., 2014 [62]	Cross-sectional 160 HD patients	dp-ucMGP	Vascular Calcification Score (Kauppila)	R = 0.17 *p* = 0.0049
Schlieper et al., 2011 [43]	188 HD patients	dp-ucMGP	Vascular calcification (X-ray and ultrasound)	No association
Schurgers et al., 2010 [104]	Prospective 107 CKD 2–5	dp-ucMGP	AOC	Associated with AOC (*p* < 0.001)
Cranenburg et al., 2009 [58]	Cross-sectional 40 HD	ucMGP	CAC	Inverse correlation (r = 0.41, *p* = 0.009)

## Abbreviations

AOC	aortic calcification
CAC	coronary artery calcification
CKD	chronic kidney disease
dp-ucMGP	not phosphorylated, undercarboxylated matrix Gla protein
HD	hemodialysis
OR	odds ratio
ucMGP	undercarboxylated matrix Gla protein
VKA	vitamin K antagonist
CF-PWV	Carotid-femoral pulse wave velocity
